# Prenatal Exposure to Arsenic Impairs Behavioral Flexibility and Cortical Structure in Mice

**DOI:** 10.3389/fnins.2016.00137

**Published:** 2016-03-31

**Authors:** Kyaw H. Aung, Chaw Kyi-Tha-Thu, Kazuhiro Sano, Kazuaki Nakamura, Akito Tanoue, Keiko Nohara, Masaki Kakeyama, Chiharu Tohyama, Shinji Tsukahara, Fumihiko Maekawa

**Affiliations:** ^1^Division of Life Science, Saitama UniversitySaitama, Japan; ^2^Department of Pharmacology, National Research Institute for Child Health and DevelopmentSetagaya, Japan; ^3^Molecular Toxicology Section, National Institute for Environmental StudiesTsukuba, Japan; ^4^Faculty of Human Sciences, Waseda UniversityTokorozawa, Japan; ^5^Faculty of Medicine, University of TsukubaTsukuba, Japan

**Keywords:** sodium arsenite, developmental neurotoxicity, behavioral impairment, neurite outgrowth, prelimbic cortex

## Abstract

Exposure to arsenic from well water in developing countries is suspected to cause developmental neurotoxicity. Although, it has been demonstrated that exposure to sodium arsenite (NaAsO_2_) suppresses neurite outgrowth of cortical neurons *in vitro*, it is largely unknown how developmental exposure to NaAsO_2_ impairs higher brain function and affects cortical histology. Here, we investigated the effect of prenatal NaAsO_2_ exposure on the behavior of mice in adulthood, and evaluated histological changes in the prelimbic cortex (PrL), which is a part of the medial prefrontal cortex that is critically involved in cognition. Drinking water with or without NaAsO_2_ (85 ppm) was provided to pregnant C3H mice from gestational days 8 to 18, and offspring of both sexes were subjected to cognitive behavioral analyses at 60 weeks of age. The brains of female offspring were subsequently harvested and used for morphometrical analyses. We found that both male and female mice prenatally exposed to NaAsO_2_ displayed an impaired adaptation to repetitive reversal tasks. In morphometrical analyses of Nissl- or Golgi-stained tissue sections, we found that NaAsO_2_ exposure was associated with a significant increase in the number of pyramidal neurons in layers V and VI of the PrL, but not other layers of the PrL. More strikingly, prenatal NaAsO_2_ exposure was associated with a significant decrease in neurite length but not dendrite spine density in all layers of the PrL. Taken together, our results indicate that prenatal exposure to NaAsO_2_ leads to behavioral inflexibility in adulthood and cortical disarrangement in the PrL might contribute to this behavioral impairment.

## Introduction

The developing brain is vulnerable to disruption by environmental factors including toxic chemical exposure. Environmental exposures may therefore account for an increase in the prevalence of neurodevelopmental and neuropsychiatric disorders including autism spectrum disorders, attention deficit hyperactivity disorders (ADHD), and learning disabilities (Grandjean and Landrigan, 2006, 2014). Recent studies suggest that *in utero* and lactational exposure to toxic chemicals affects the development of the brain. For example, exposure to inorganic lead, methylmercury, and polychlorinated biphenyls during gestation and early childhood are associated with the prevalence of mental retardation, cerebral palsy, and ADHD in children (Grandjean and Landrigan, 2006; Bisen-Hersh et al., 2014). These studies indicate that early life environmental exposures play a role in the etiology of neurodevelopmental disorders.

It has been long suspected that arsenic exposure can lead to developmental neurotoxicity. More than 200 million people worldwide have been estimated to be chronically exposed to arsenic in drinking water at concentrations above the World Health Organization (WHO) recommended safety limit of 10 μg/L (WHO, 2008). A large number of epidemiological studies have demonstrated that chronic exposure to arsenic produces peripheral neuropathies and decreases cognitive performance in children such as lowered memory and intelligence quotient scores on standardized tests (Rocha-Amador et al., 2007; Rosado et al., 2007; Wasserman et al., 2007), which are indicative of higher brain function deficits. Additionally, follow-up studies on victims of arsenic poisoning from the Morinaga formula incident in Japan revealed an association between oral exposure to arsenic during infancy and various brain disorders, including mental retardation and epilepsy (Dakeishi et al., 2006). These studies suggest that early life arsenic exposure can affect higher brain function later in life. This notion is supported by some studies in animal models. For example, exposure to low level arsenic in maternal drinking water throughout gestational and lactational period increased indices of anxiety in mouse offspring during a novel object exploration task (Martinez-Finley et al., 2009). Moreover, few behavioral deficits such as an increase in pivoting, a type of abnormal gait behavior, was observed in younger mouse offspring following a short period of gestational exposure to arsenic (Colomina et al., 1996).

Arsenic exposure could produce behavioral changes through effects on the developing brain directly since arsenic freely crosses the fetus-placenta and blood-brain barrier in human (Willhite and Ferm, 1984; Hirner and Rettenmeier, 2010). *In vitro* and *in vivo* experimental models have been used to elucidate how arsenic exposure impairs higher brain function. A previous study showed that sodium arsenite (NaAsO_2_) exposure produces both apoptotic and necrotic cell death in developing brain cells in rat (Chattopadhyay et al., 2002). Our *in vitro* studies have shown that NaAsO_2_ exposure induces apoptotic cell death and inhibits neuritogenesis (Koike-Kuroda et al., 2010; Aung et al., 2013). The inhibitory effect of NaAsO_2_ on neuritogenesis is in part result from alterations in cytoskeletal components (Aung et al., 2013) and the downregulation of AMPA receptors, which are known to regulate the expression of cytoskeletal proteins (Maekawa et al., 2013). In animal studies, embryonic arsenic exposure produces neural tube defects, increase neuronal apoptosis, disrupt neural outgrowth, and reduce overall head size in both mouse and zebrafish models (Chaineau et al., 1990; Li et al., 2009). Further, it has been reported that arsenic exposure in rats from gestation throughout lactation and development until the age of 4 month alters morphology of nerve fibers and axon in the corpus striatum (Rios et al., 2009). These studies indicate that structural changes of brain such as neural network formation might contribute to the impairment of higher brain function following early life exposure to NaAsO_2_. However, the precise mechanism by which developmental arsenic exposure produces impairments in higher brain function remains largely unknown.

Executive function such as planning, goal-directed action, and behavioral flexibility are core units of higher brain function, and impairment of these functions has been observed in a variety of neurodevelopmental disorders (Valencia et al., 1992; Kipp, 2005; Hill and Bird, 2006). To evaluate these executive processes in mice, a model of behavioral flexibility was recently established using the IntelliCage system, which is a fully automated behavioral testing apparatus for mice under group-housing conditions (Endo et al., 2011, 2012). This testing apparatus allows for the comprehensive and reproducible evaluation of behavioral flexibility. In humans, the brain areas responsible for executive function of goal-directed actions and behavioral flexibility are located in the medial prefrontal cortex (Yan et al., 2015). Several studies have shown that the prelimbic cortex (PrL), a part of the medial prefrontal cortex, is critically involved in a variety of cognitive and executive processes (Dalley et al., 2004; Marquis et al., 2007; Ragozzino, 2007). It has been also reported that the PrL is affected by exposure to chemicals, such as methylmercury, lead, and dioxin, and that exposure-associated impairments in the PrL are associated with decreased executive function in rodents (Ferraro et al., 2009; Tomasini et al., 2012). Accordingly, we decided to investigate the structure of the PrL as a possible target of arsenic-induced brain impairment.

In the present study, we used a fully automated behavioral analysis system to investigate the effects of prenatal NaAsO_2_ exposure on murine behavioral flexibility in adulthood, and then analyzed the morphology of neuronal cells in these animals in order to determine how early life NaAsO_2_ exposure produces neurotoxicity.

## Materials and methods

### Animals and NaAsO_2_ exposure

Pregnant C3H mice were purchased from JAPAN SLC (Shizuoka, Japan) and housed on a 12-h light/dark cycle at a temperature of 24 ± 1°C with free access to water and food. From gestational days 8–18, pregnant females were given *ad libitum* access to regular water or water containing 85 parts per million (ppm) NaAsO_2_ (equivalent to 85 mg/L). To examine the water consumption of pregnant dams, the weight of water bottle for each dam was measured before and after providing *ad libitum* access to water. Pregnant dams tolerated the dose of NaAsO_2_ at 85 ppm, and no obvious effects on maternal toxicity or teratogenicity were observed.

The pups were weaned at post-natal day 21 and housed under the same conditions as the dams. The number and sex of pups born from dams were then measured. At 60 weeks of age, control and NaAsO_2_-exposed offspring were prepared for behavioral flexibility testing using the IntelliCage system (TSE Systems GmbH, Bad Homburg, Germany). Mice were randomly selected per dams (number of dams: control = 6 and NaAsO_2_ = 9) to minimize the litter effects. After selecting mice, they were lightly anesthetized with diethyl ether and subcutaneously implanted with a glass-covered transponder. Each transponder had a unique ID code for radiation frequency identification (RFID) for use with the IntelliCage system. Males and females were separately tested using different IntelliCage apparatuses. The control and NaAsO_2_-exposed groups includes 9 mice per group for females and 6 or 10 mice per group for males. All procedures were approved by the Institutional Animal Care and Use Committee of the National Institute for Environmental Studies (NIES) and conducted strictly in accordance with NIES guidelines.

### Intellicage apparatus

**Figure 1:**
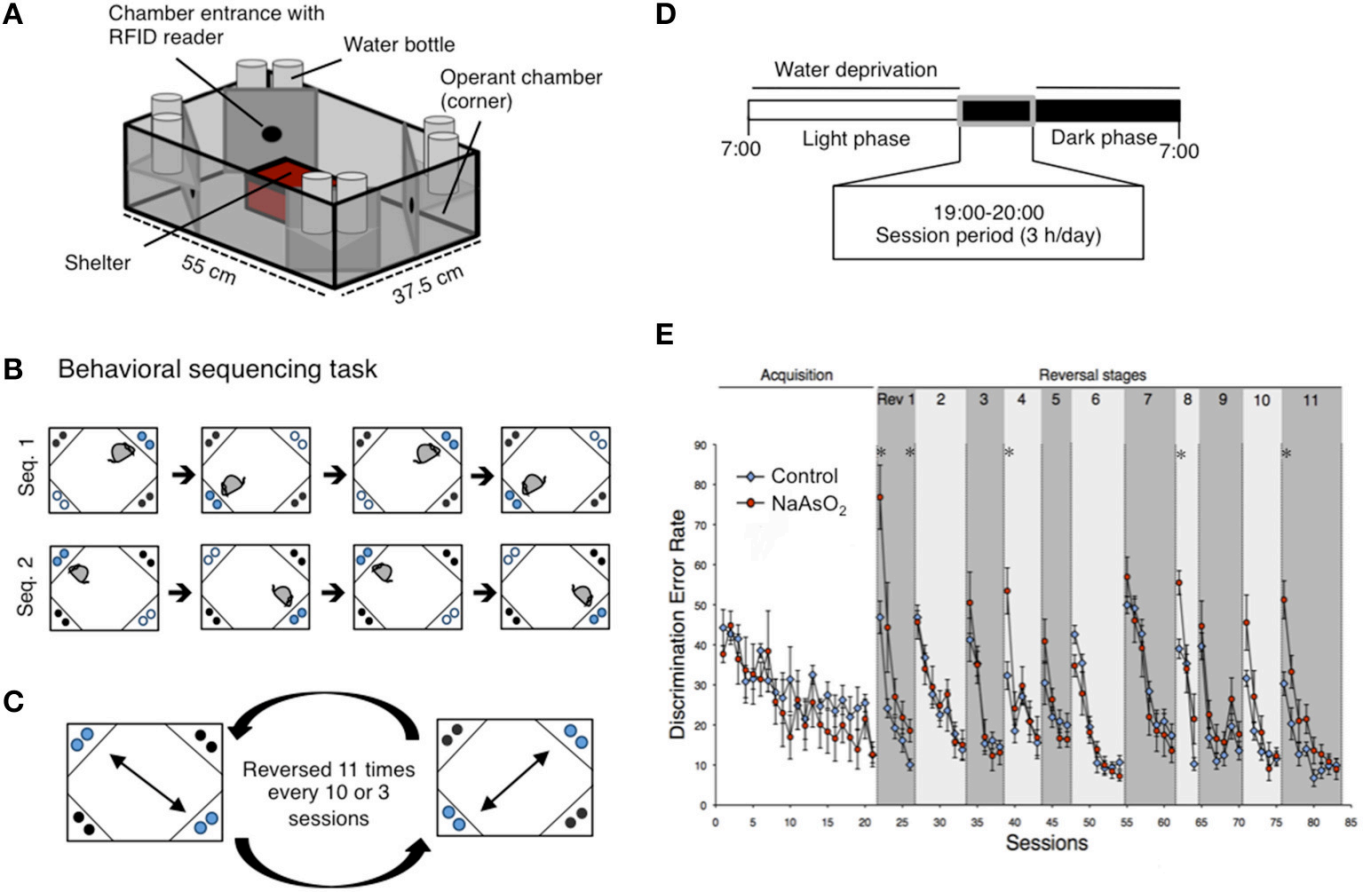
**Impaired behavioral flexibility in adult mice born to dams that were administered NaAsO_**2**_ during gestation. (A)** Overview of an IntelliCage apparatus. **(B)** Behavioral sequencing task. Mice were allowed to make an access to water as a reward for 4 s during their visits at an active reward corner (blue circle). The location of the active reward corner was automatically alternated to a diagonally opposed corner each time the mouse received a reward. Therefore, mice obtained rewards continuously by acquiring a behavioral sequence (alternating between the two reward corners). A visit to a never-rewarded corner (black circle) was counted as a discrimination error. **(C)** Serial reversal task. For each mouse, the assigned spatial patterns of the rewarded corners (sequence 1 or sequence 2) were reversed 11 times every 3 or 8 sessions. **(D)** Timeline of the experiment for each day. **(E)** Learning performance of control or NaAsO_2_-exposed female mice in the behavioral flexibility test. Discrimination error rates (the number of discrimination errors in the first 100 corner visits of a session) are expressed as mean ± SEM (control group *n* = 8, NaAsO_2_-exposed group *n* = 6). An asterisk indicates a statistical difference (*p* < 0.05) from the control group.

The IntelliCage is a computer-based, fully-automated testing apparatus that can be used to monitor the spontaneous and cognitive behaviors of group-housed RFID-tagged mice in a large home cage (Figure 1A). Briefly, a large standard plastic cage (55 × 37.5 × 20.5 cm^3^) was equipped with four triangular operant learning chambers (hereafter referred to as corners) (15 × 15 × 21 cm^3^) that fit into each corner of the cage. RFID readers and other sensors allowed the simultaneous monitoring of up to 16 transponder-tagged mice living in the same cage. Mice were allowed to enter each corner (hereafter referred to as a “corner visit”) through a short, narrow tunnel that functioned as an RFID antenna. Only one mouse was able to enter a given corner at any one time due to the limited size of the tunnel. In the inner space of each corner was equipped with two nose poke holes that were monitored via an infrared beam-break response detector. Nose poke behavior triggered to open a motorized gate access to a water bottle nipple. For each behavioral event (corner visit, nose poke, and licking), mouse ID and corner ID were automatically recorded through the RFID readers, infrared sensors, and lickometers.

### Intellicage test procedures

#### Acclimation

The control and NaAsO_2_-exposed male or female mice at 67 weeks of age were separately introduced to IntelliCage apparatuses on the same day. The number of mice in each IntelliCage apparatus was counterbalanced within groups. Acclimation and behavioral tests were then conducted according to test procedures. In acclimation phase 1 (3 days), the motorized gates controlling access to water bottle nipples were kept open in all 4 corners; thus, mice were allowed to drink water in each corner *ad libitum*. In acclimation phase 2 (1 day), the mice were trained to perform the nose poke task. Initially, all motorized gates were closed and mice were only given access to water bottle nipples after a nose poke event. The gate remained open for 4 sec following each nose poke, and water was available through the nose poke task for 24 h. In acclimation phase 3 (5 days), mice were only given the opportunity to gain access to water through the nose poke task for a 3-h period (19:00–22:00) per day. During acclimation phase, four female mice (one from control group and three from NaAsO_2_-exposed group) were not able to learn how to access water drinkable corners, and such mice were not used in the following behavioral tasks.

#### Behavioral sequencing task

The behavioral flexibility test, also referred to as the behavioral sequencing task, was composed of an acquisition phase and a serial reversal task phase. The acquisition phase consisted of 11 or 21 sessions of the behavioral sequencing task (Figure 1B) and the serial reversal task phase consisted of repetitions of a reversal task (Rev. 1−11, Figure 1C). Water-deprived mice had 4 sec of access to water as a reward when they visited designated corners during a daily 3-h test session (19:00−22:00; Figure 1D). A total of 67 sessions for male mice and 83 sessions for female mice were conducted. In each session, mice were rewarded continuously if they alternated visits between two particular diagonally opposed corners (Figure 1C). The diagonal pair of corners was either active or inactive in a mutually exclusive manner, meaning that there was always one active reward corner, one inactive reward corner, and two never-rewarded corners. Mice were able to open the gate in an active corner by nose poke, and the gate remained open for 4 s to permit drinking. After the reward period, the corner instantly became inactive, and this signal was synchronized with the activation of the diagonally opposed corner. The alteration of corner assignments was controlled for each mouse independently by the IntelliCage software. Thus, the mice had to alternate between two diagonally opposed reward corners in order to acquire rewards continuously. A visit to either of the two never-rewarded corners was regarded as a discrimination error. The number of discrimination errors within the first 100 visits in each session provided a discrimination error rate that was used to analyze inter-session learning performance.

### Histological staining

After the last session of behavioral experiments, the same female mice were immediately sacrificed for morphometrical analysis of neuronal cells, while male mice were used for gene expression analyses (not described in this study). Mice were deeply anesthetized with sodium pentobarbital (60 mg/kg) and brains were harvested for analysis. Brains were histologically processed using the FD Rapid GolgiStain Kit (FD NeuroTechnologies, Ellicot City, MD, USA). Briefly, brains were rinsed with distilled water, immersed in 5 mL of equal parts Solution A and Solution B at room temperature, and stored for 2 weeks in the dark. Storage solution was replaced with fresh solution on the second day. Tissues were next immersed in Solution C at 4°C for at least 48 h. Solution C was replaced with fresh solution on the second day. Samples were then quickly frozen at −70°C and stored at −20°C until use. Coronal brain sections (60 μm) were cut using a cryostat (Leica CM1900; Leica Microsystems, Wetzlar, Germany), where the temperature of chamber and specimen head were set to −22°C and −23°C, respectively. Brain sections were mounted on gelatin-coated glass slides and allowed to dry at room temperature. Sections were then stained with a solution of 1 part Solution D, 1 part Solution E, and 2 parts distilled water for 10 min at room temperature. Golgi-stained brain sections were rinsed in distilled water twice for 4 min each and counterstained with 0.1% Cresyl Fast Violet solution. The Golgi- and Cresyl Fast Violet-stained sections were used for stereological analysis. The group average number of neurons and glial cells, length of neurites, and density of dendritic spines were calculated from five or seven brains of mice, which was randomly selected and blind from behavioral data, for control and NaAsO_2_-exposed group, respectively.

### Stereological analysis of the length of neurites and the number of neuronal cells

**Figure 2:**
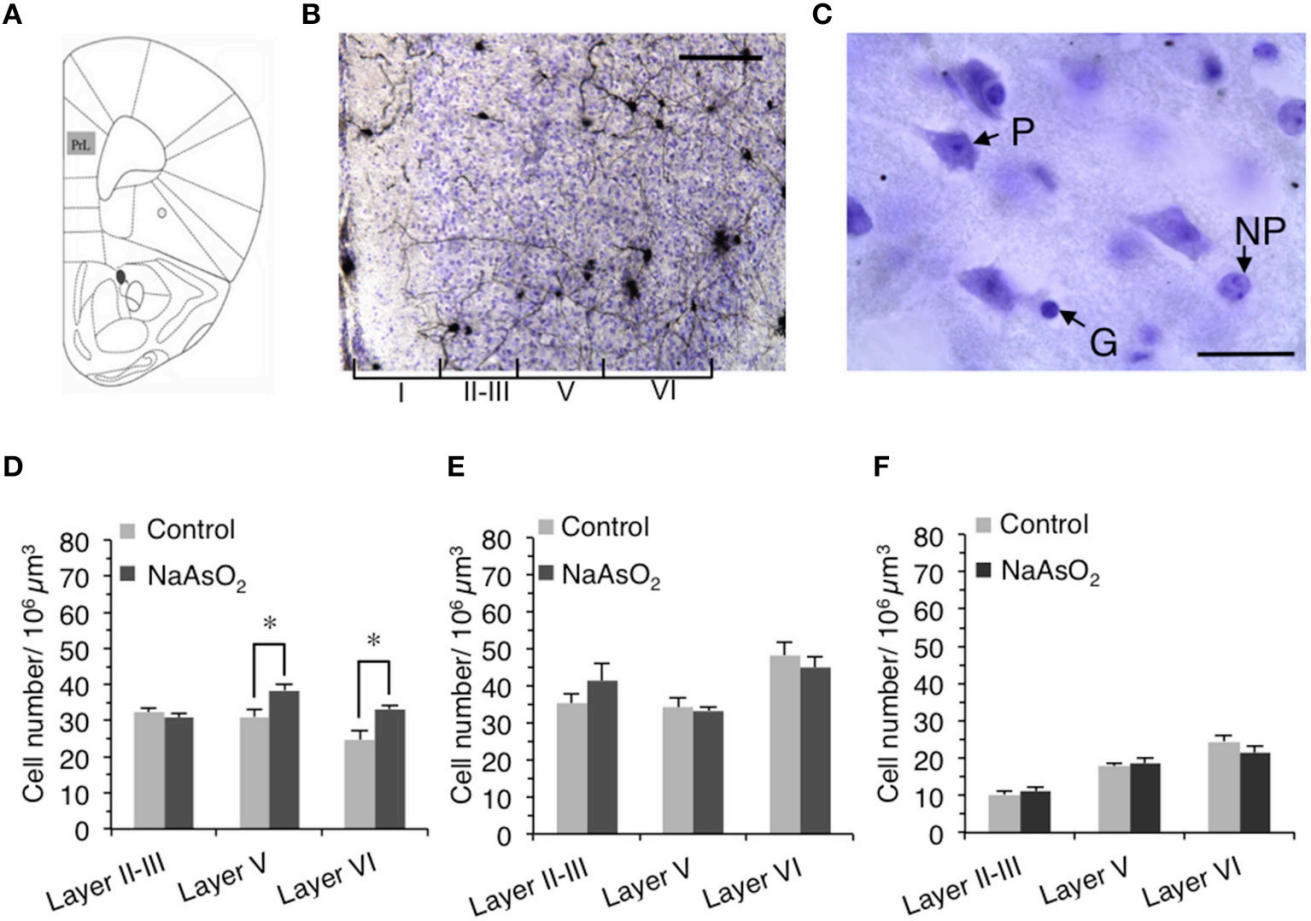
**Alteration in the number of neuronal cells in the PrL of adult female mice born to dams that were administered NaAsO_**2**_. (A)** Illustration of the region of interest, the PrL, adapted from a mouse brain atlas (Franklin and Paxinos, 2008). **(B)** Representative photomicrograph of the PrL in Golgi- and Cresyl Fast Violet-stained coronal brain sections. The Greek numbered (I–VI) regions indicate the cortical layers of the PrL. Scale bar = 20 μm. **(C)** Photomicrographs of the PrL at high magnification showing a Cresyl Fast Violet-stained pyramidal neuron (P), a non-pyramidal neuron (NP), and a glial cell (G). The length of scale bar indicates 5 μm. The number of pyramidal neurons **(D)**, non-pyramidal neurons **(E)**, and glial cells **(F)** in the PrL. Values are expressed as mean ± SEM (control group *n* = 7, NaAsO_2_-exposed groups *n* = 5). An asterisk indicates *p* < 0.05 vs. the control group.

The length of neurites and the number of neurons and glial cells in the PrL were measured using StereoInvestigator software (MicroBrightField (MBF) Bioscience, Williston, VT., USA) and a light microscope (DM5000B; Leica Microsystems) connected to a CCD camera. The boundaries between the PrL and the infralimbic cortex (IL) and between the PrL and the dorsal anterior cingulate (ACd) were determined by observing differences in cell size and density in the cortical layers of Cresyl Fast Violet-stained brain sections (Van De Werd et al., 2010). The rostrocaudal level of the PrL was determined by referring to an atlas of the mouse brain (Franklin and Paxinos, 2008; Figure 2A).

**Figure 3:**
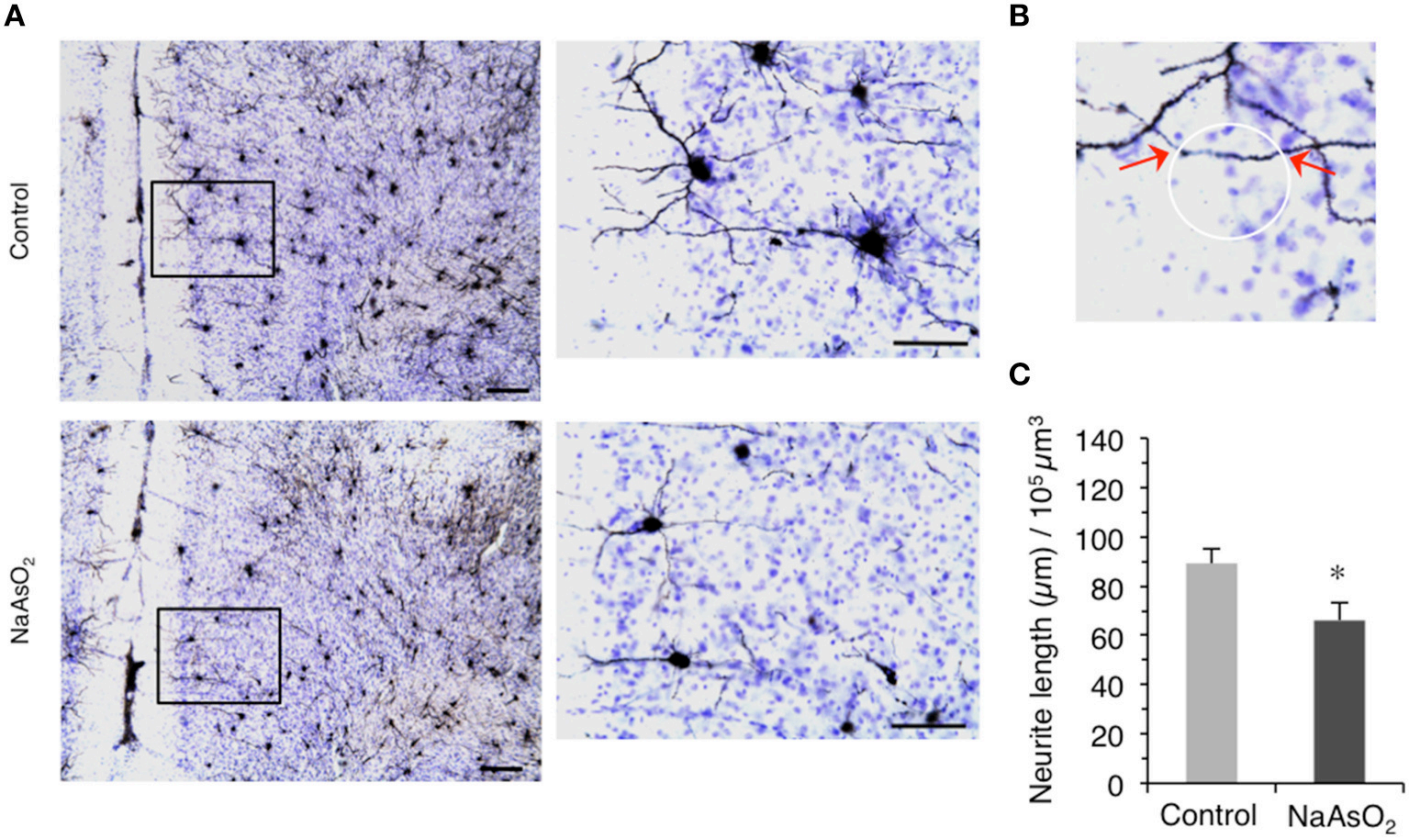
**Altered morphology of neurites in the PrL of adult female mice born to dams that were administered NaAsO_**2**_ during gestation. (A)** Representative photomicrographs of the PrL in Golgi- and Cresyl Fast Violet-stained coronal brain sections from female mice with or without prenatal exposure to NaAsO_2_. The rectangular area was magnified and shown in the corresponding right panel. Bars = 20 μm (low magnification) and 10 μm (high magnification). **(B)** Demonstrative photomicrograph for the intersection points (indicated by red arrows) between a virtual sphere “Space Ball” (white circle) and Gogli-stained neurite. **(C)** Estimated neurite lengths in Golgi-stained PrL neurons as measured by the Space Ball method. Values (mean ± SEM) were normalized to the estimated volume of the selected PrL region (control group *n* = 7, NaAsO_2_-exposed group *n* = 5). An asterisk indicates *p* < 0.05 vs. the control group.

The length of neurites on Golgi-stained neurons in the left PrL was measured using the Space Ball probe utility of the Stereo Investigator software. The contours of the PrL in each brain section were drawn using a 5X objective lens magnification according to the criteria mentioned above. We set grid sizes of 150 × 200 μm, and used a sphere with a 40-μm radius and a highest top guard zone of 2.5 μm for the quantification of neurite length. The intersectional points between Golgi-stained neurites and the spherical line (Figure 3B) were counted in three consecutive sections of the PrL using a 100X oil immersion objective lens magnification, and the number of intersection points was used to compute the estimated length of Golgi-stained neurite in the selected region of the PrL for each mice (Mouton et al., 2002). The estimated length of Golgi-stained neurite was then normalized by dividing it by the estimated volume of the selected region in each animal. The coefficient of error (Gundersen, *m* = 1) for the estimation of neurite length was 0.05–0.09 for each animal.

The optical fractionator method was used to measure the number of Cresyl Fast Violet-stained neurons (pyramidal and non-pyramidal neurons) and glial cells in three different cortical layers (layer II−III, V, and VI) of the left PrL in accordance with the system work flow of the Stereo Investigator software. Since cortical layer IV is completely disappeared in the mice PrL (Van De Werd et al., 2010), it was not included in this analysis. The cortical layer boundaries were distinguished based on differences in cell size and density in Cresyl Fast Violet-stained brain sections (Figure 2B). The contours for layers II–III, V, and VI were drawn in each section using a 5X objective lens magnification and a frame size of 30 × 30 μm within the grid size of 150 × 150 μm. The height of the optical dissector was 40 μm and the top guard zone was 2.5 μm. Cell numbers were counted manually using a 100X oil immersion objective lens magnification. The setting for cell counting was sufficient to generate a coefficient of error (Gundersen, *m* = 1) of 0.05–0.06. The estimated number of pyramidal neurons, non-pyramidal neurons, and glial cells in each layer of the PrL was normalized by dividing each number by the estimated volume of its respective layer. The morphological criteria used to identify neuronal and glial cells observed in Cresyl Fast Violet-stained brain sections have been previously reported (Tsukahara et al., 2011). To distinguish pyramidal neurons from non-pyramidal neurons, the following criteria were used: (1) the cell bodies of pyramidal neurons exhibited a characteristic triangular shape with a single large apical dendrite extending vertically toward the pial surface, (2) non-pyramidal neurons were identified by the absence of the preceding criteria and exhibited a relatively smaller cell body size than that of pyramidal neurons (Figure 2C).

### Imaging and analysis of dendritic spine morphology

The dendritic segments of Golgi-stained pyramidal neurons were used in morphometrical analyses. Sequential z-series images of dendritic segments were taken every 0.4 μm with an oil immersion lens (Plan Apo VC 100X, Numerical Aperture 1.40, Oil; Nikon, Tokyo, Japan) and a BioRevo 9000 microscope (Keyence Co., Osaka, Japan). The applied zoom factor (1.5X) provided images with 0.14 μm/pixel resolution. Images were then deconvoluted using Keyence BZ II Analyzer software (Keyence) and constructed into three-dimensional images using ImageJ software (National Institutes of Health, Bethesda, MD, USA) for analyses of dendritic spine morphology. The density and head diameter of dendritic spines were analyzed using Spiso-3D automated dendritic spine analysis software, which has an equivalent capacity to Neurolucida (MBF Bioscience, USA) (Mukai et al., 2011). The primary basilar dendritic segments of Golgi-stained pyramidal neurons, lying between 10 and 100 μm from the soma, were used to analyze the morphology of dendritic spines. For each cortical layer (layer II−III, V, or VI), 30−45 dendritic segments were analyzed per experimental group. Spine density was calculated from the number of spines existing on the total length of 40−100 μm dendritic segments. To examine spine morphological changes in response to prenatal NaAsO_2_ exposure, the diameter of the spine head was classified into three categories: (1) small-head spines with a diameter of 0.2−0.4 μm, (2) middle-head spines with a diameter of 0.4−0.5 μm, and (3) large-head spines with a diameter of 0.5−1 μm.

### Statistical analysis

Changes in mouse behavioral flexibility were analyzed using the non-parametric Mann Whitney *U*-test with R software (The R Foundation for Statistical Computing, Vienna, Austria) because the sample size of each group for each session was relatively small and it didn't follow a normal distribution. Morphometrical and other general assessments (including body weight, number of pups, and water intake) were analyzed with the parametric Student's unpaired *t*-test with Welch's correction with Prism software (GraphPad Software, La Jolla, CA, USA). Statistical differences were evaluated between the control and NaAsO_2_-exposed groups. *P* ≤ 0.05 were considered to be statistically significant.

## Results

### Maternal and embryonic toxicity

No dams were found to develop significant abnormalities in general health parameters including the body weight gain of the dams during pregnancy (Figure S1) and maternal death. In addition, there were no differences in the number of live pups between the control and NaAsO_2_-exposed groups (Figure S2).

### Basal activity levels of offspring

No toxic effects of prenatal NaAsO_2_ exposure on body weight gain and blood glucose level of offspring were observed (Figure S3). In addition, there were no apparent differences in most of basal activity indices of the offspring in the acclimation phase of the behavioral flexibility test were observed between the two groups, except significant increase in duration of nose poke in NaAsO_2_-exposed female mice (Tables S1, S2).

### Impaired behavioral flexibility in NaAsO_2_-exposed mice

Behavioral flexibility was examined by evaluating the number of incorrectly visiting the two never-rewarding corner within the first 100 visits of a given test session (discrimination error rate). In acquisition phase of the behavioral sequencing task, mice were imposed to discriminate rewarded corners from never-rewarded corners with acquirement of shuttling behavior between the two distantly positioned rewarded corners to obtain water continuously (Figure 1B). No apparent differences in the acquisition of the behavioral sequencing tasks were observed between the control and NaAsO_2_-exposed groups of both sexes (Figure 1E, Figure S4). However, a delay in acquiring the behavioral sequencing tasks was observed in both the control and NaAsO_2_-exposed females. While the discrimination error rate of both the control and NaAsO_2_-exposed males was significantly decreased to approximately 10% by session 11 (Figure S4), the discrimination error rate of both groups of females was decreased to approximately 15% by session 21 (Figure 1E). It indicates that female mice took longer time to be able to adapt the behavioral sequencing task than that taken by male mice regardless of prenatal exposure to NaAsO_2_.

In the subsequent serial reversal task, the discrimination error rate for the control and NaAsO_2_-exposed groups of both female and male mice was elevated in the first session of each reversal phase (Rev 1−11; Figure 1E, Figure S4), indicating that each group of both male and female mice properly acquired the behavioral sequence assigned in the previous phase. However, in NaAsO_2_-exposed female mice, the discrimination error rate was significantly higher than that of the control mice in the first session of reversals 1, 4, 8, and 11, and in the fifth session of reversal 1 (Figure 1E). These results suggest that NaAsO_2_-exposed female mice are impaired in the initial adaptation process of reversal learning. Nevertheless, the increased discrimination error rate in the first session of reversals was significantly reduced in subsequent reversal phase sessions of both groups of female mice (Figure 1E), demonstrating a day-to-day improvement in the adaptive behavior in female mice. In male mice, tendency of overall increases in discrimination error rate between the control and NaAO_2_-exposed groups was observed. Significant increases were observed in the second session of reversals 5 and 11 (Figure S4), whereas significant decrease in discrimination error rate was found in first session of Rev 6. These results suggest that NaAsO_2_-exposed male mice showed impairment in adaptation to reversals, but the degree of impairment in males might not be severe compared to that in females.

### Alteration in the number of neurons and glial cells in the PrL of NaAsO_2_-exposed mice

To determine whether NaAsO_2_ exposure-related behavioral alterations in mice are associated with changes in brain histology, the number of Cresyl Fast Violet-stained pyramidal neurons, non-pyramidal neurons, and glial cells were measured in three different layers (layer II−III, V, and VI) of the PrL (Figures 2B,C). Stereological analysis revealed that the number of pyramidal neurons in layers V and VI but not in layer II−III of the PrL was significantly (*p* < 0.05) increased in the NaAsO_2_-exposed group as compared to the control group (Figure 2D). No significant differences in the number of non-pyramidal neurons and glial cells were observed between the control and *NaAsO*_2_*-exposed* groups in any observed layer of the PrL (Figures 2E,F).

### Alteration of the morphology of neurites in the PrL of NaAsO_2_-exposed mice

We next evaluated the morphology of neurites on Golgi-stained neurons in the PrL. A reduction in the length of neurites on Golgi-stained neurons in the PrL was observed in NaAsO_2_-exposed mice as compared to neurites in the control group (Figure 3A). Space Ball probe analysis indicated that NaAsO_2_ exposure was associated with a significant (*p* < 0.05) decrease in the length of neurites in Golgi-stained neurons of the PrL as compared to the control group (Figure 3C).

### The density and morphology of dendritic spines of pyramidal neurons in the PrL of NaAsO_2_-exposed mice

**Figure 4:**
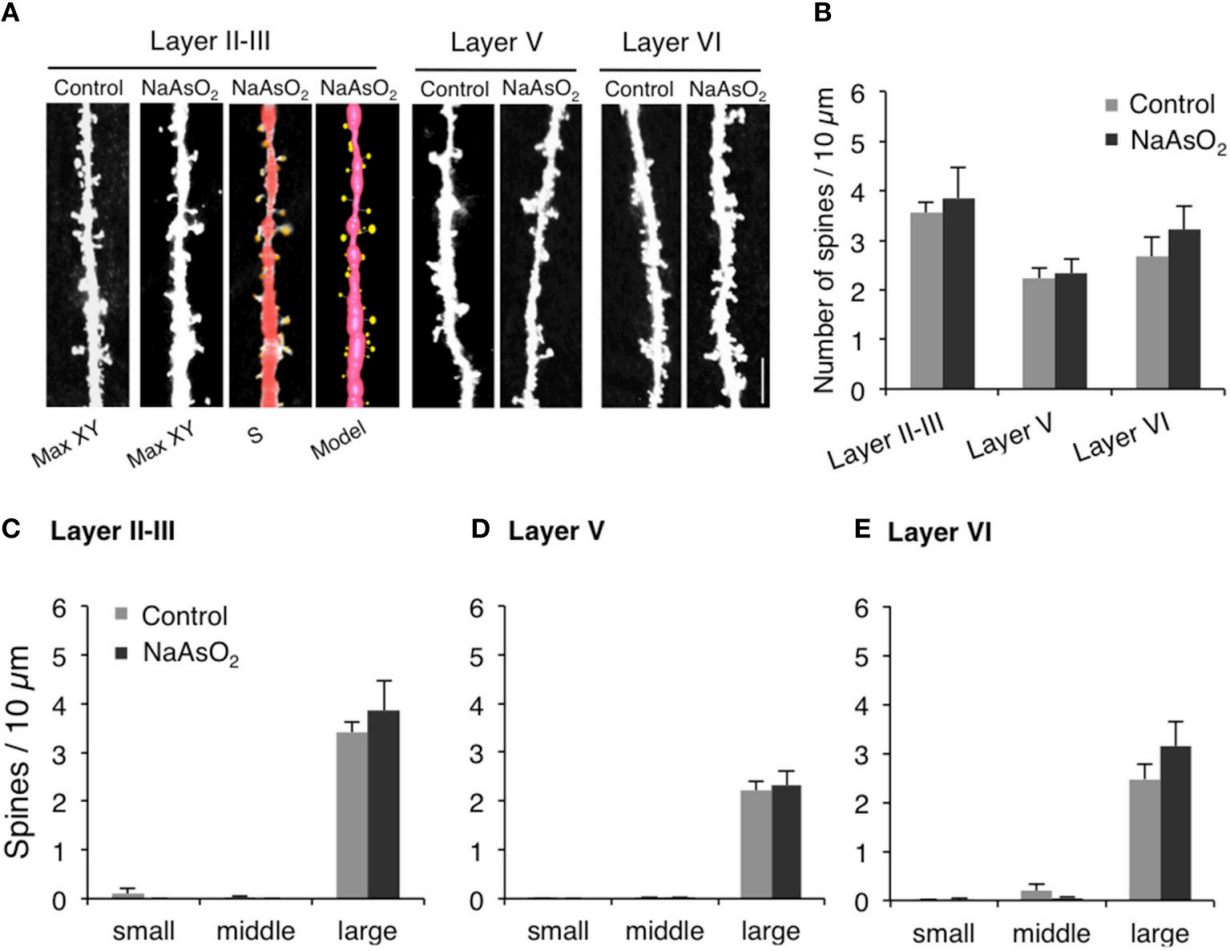
**The density and the morphology of dendritic spines in the PrL of adult female mice born from dams exposed to NaAsO_**2**_ during gestation**. Dendritic spines were analyzed along the primary basilar dendrites of Golgi-stained pyramidal neurons in three different cortical layers (layer II−III, V, and VI) of PrL. **(A)** Representative photomicrographs of dendritic spines with maximal intensity projections onto the XY plane from Z-series images (Max-XY) for the control group (Control) and NaAsO_2_-exposed group. Photomicrographs analyzed by Spiso-3D (S) and three-dimensional models (Model) are also shown for the analysis of dendritic spines in the layer II−III of the NaAsO_2_-exposed group. **(B)** Effect of NaAsO_2_ exposure on the total dendritic spine density of pyramidal neurons in three different cortical layers. Effect of NaAsO_2_ exposure on the density of three subtypes of dendritic spines, small-head spines (small), middle-head spines (middle), and large-head spines (large), in three cortical layers of the PrL: Layer II−III **(C)**, Layer V **(D)**, and Layer VI **(E)**. The density of dendritic spines is expressed as the number of spines per 10 μm of dendrite. A total 500−1200 spines from 30 to 45 dendritic segments of 30−45 neurons were analyzed for each cortical layer. Values are expressed as mean ± SEM (control group *n* = 7, NaAsO_2_-exposed group *n* = 5).

The density and head diameter of dendritic spines from Golgi-stained pyramidal neurons were measured in three different cortical layers (layer II−III, V, and VI) of the PrL (Figure 4A). The total density of dendritic spines in pyramidal neurons was not significantly different between control mice and NaAsO_2_-exposed mice in any observed layer of the PrL (Figure 4B). Spine head diameters were also not significantly different between the control and NaAsO_2_-exposed groups in any observed layer of the PrL (Figures 4C–E).

## Discussion

In the present study, we investigated the effect of prenatal NaAsO_2_ exposure on the behavioral flexibility/reversal learning of adult mice using the IntelliCage system, which is an efficient tool for monitoring multiple aspects of cognitive behavior in a social environment (Endo et al., 2012; Benner et al., 2015). The core finding of our work is that exposure of dams to NaAsO_2_ produces behavioral inflexibility to reversal learning and abnormal formation of the PrL in adult offspring. These findings suggest that behavioral impairments caused by NaAsO_2_ exposure are associated with structural changes of brain, particularly in the PrL cortical region.

Here, we provided pregnant female mice with drinking water that contained 85 ppm NaAsO_2_ during a critical period of embryonic brain development (gestational day 8–18). A series of studies by Colomina et al. evaluated the effect of NaAsO_2_ exposure on development of nervous system. Exposure to NaAsO_2_ at 10 mg/kg/day throughout gestational day 15–18 delayed neurodevelopmental indices such as eye opening in female offspring (Colomina et al., 1997). Furthermore, they have shown that single NaAsO_2_ exposure at 30 mg/kg induces deficit in neuromotor development (Colomina et al., 1996). In accordance with these studies, we considered that providing NaAsO_2_ about 10 mg/kg to pregnant mice during gestation might induce higher brain function deficits in offspring. It has been reported that mice drink around 5 ml of water daily (Bachmanov et al., 2002). However, our own measurement showed that a 25–35 g pregnant mouse drink about 3.5 ml of water daily when the mouse is provided with NaAsO_2_-containing water (Figure S5). Based on these estimates, a 25–35 g dam consumes 0.39 mg NaAsO_2_/day (which is equivalent to 8.5–12 mg/kg/day) when dams are provided with drinking water containing 85 ppm NaAsO_2_. The selected NaAsO_2_ dose used in the current study did not produce any obvious maternal toxicity or embryonic toxicity (Figures S1, S2), which is consistent with the previous studies (Rodriguez et al., 2002; Waalkes et al., 2003; Markowski et al., 2012).

Behavioral flexibility describes the ability of an organism to adapt to a changing environment. Behavioral flexibility occurs in many kinds of animal, such as mice, rat, and monkey, and is often assessed using rule-shift learning task paradigms that include a sequencing reversal task. In this study, we assessed the behavioral flexibility of control or NaAsO_2_-exposed female mice using an altered action-outcome contingency paradigm during inter-sessions, and inter-reversal stages that included a serial reversal task (Endo et al., 2012). In the inter-session analysis, day-to-day improvements in adaptive behavior, as observed in a decreasing trend of discrimination error rate in each reversal phase, were clearly observed not only in control but also arsenite-exposed groups. However, NaAsO_2_-exposed mice demonstrated a lower degree of achievement in reversal learning than the control group. It may be inferred that the repetition of reversal learning lead to difficultly in re-acquiring reversal learning for the NaAsO_2_-exposed group. Such effects of arsenic have been reported in adulthood exposure. A series of studies have demonstrated that arsenic exposure in adult mice produces an increase in the number of errors in an egocentric task (Rodriguez et al., 2001, 2002). Our present findings suggest that NaAsO_2_ exposure in early life also produces behavioral impairments in learning function in mice. The first session of each reversal stage in the serial reversal-learning task tests the ability of mice to adapt to a changing task, because in this first session, mice must alter their behavioral sequence in order to receive a reward. Mice in the control group adapted to new behavioral sequences after a series of reversals, whereas mice in the NaAsO_2_-exposed group did not adequately adapt to changing tasks (Figure 1E) that was likely indicative of behavioral inflexibility.

We also revealed a possible link between behavioral alterations and structural changes in the PrL cortical region. The PrL is known to be involved in the regulation of cognitive and executive processes (Dalley et al., 2004; Marquis et al., 2007; Ragozzino, 2007). It has been demonstrated that the PrL plays a fundamental role in behavioral flexibility. For example, patients who have the frontal lobe (including PrL) damage show impaired adaptation to changes in reinforcement contingencies in spite of the fact that these patients can acquire novel skills or adopt new rules with relative ease (Owen et al., 1993). It has also demonstrated that either lesion or inactivation of the PrL impairs behavioral flexibility in rodents (Ragozzino et al., 1999). Accordingly, structural changes in the PrL can contribute to the impairment of behavioral flexibility. NaAsO_2_ is known to produce neurotoxicity by inducing apoptotic cell death (Wong et al., 2005; Keim et al., 2012) and/or cellular necrosis (Chattopadhyay et al., 2002; Yang et al., 2003). Therefore, in the present study, we measured the number of neurons and glial cells in the PrL in order to determine whether cell viability was affected by prenatal NaAsO_2_ exposure. Contrary to our expectations, these morphometrical analyses revealed that NaAsO_2_ exposure increased the number of pyramidal neurons in layers V and VI of the PrL (Figure 2D). Our previous *in vitro* work showed that a high concentration of NaAsO_2_ (2 μM) reduced the viability of mouse primary cortical neurons, but that a low concentration of NaAsO_2_ (0.5 μM) conversely increased cell viability and promoted cellular proliferation (Maekawa et al., 2013). It suggests that the concentration of NaAsO_2_ (85 ppm) used in the present study reflect the low concentration of NaAsO_2_ exposure resulting in the increase in the number of pyramidal neurons in the present. On the other hand, the number of non-pyramidal neurons and glial cells was not affected by NaAsO_2_ exposure in the present study. The difference between the effect of NaAsO_2_ exposure on pyramidal and non-pyramidal cells (Figures 2D,E) could be due to differences in the timing of neurogenesis and neuronal migration. The majority of cerebral cortical neurons are generated during embryonic day 11–17 in mouse (Price and Lotto, 1996; Price et al., 1997), whereas neurogenesis for each cortical layer is not simultaneous and occurs with variable timing (Finlay and Darlington, 1995). Additionally, pyramidal neurons are generated from the ventricular zone and migrate through the cortical layers radially, while interneurons including non-pyramidal neurons are generated from the ganglionic eminence and migrate tangentially (Nadarajah et al., 2003). Therefore, the timing of generation and migration of pyramidal neurons is different from that of non-pyramidal neurons, and these differences may reflect the layer-specific and cell type-specific effects of NaAsO_2_ on the number of neurons in the PrL observed in the present study. Several studies have already shown that chemical exposures affect neuronal migration by disrupting the inside-out pattern of migration (Kakita et al., 2002; Schreiber et al., 2010). Taken together, the generation and migration of neurons may be at least partially affected by prenatal NaAsO_2_ exposure, although the mechanisms by which NaAsO_2_ exposure specifically increases the number of pyramidal neurons in a layer-dependent manner has not yet been identified. Regarding glial cells, morphological or functional changes have been shown to occur at higher NaAsO_2_ concentrations than those, which affect the morphology of neurons (Wang et al., 2012). Therefore, the observed lack of effect of NaAsO_2_ exposure on the number of glial cells in this study was expected, and may be due to an insufficient level of prenatal NaAsO_2_ exposure.

Another critical finding of our study is that behavioral inflexibility is clearly associated with structural changes in PrL neurons. We previously demonstrated that NaAsO_2_ disrupts neuritogenesis in primary cultured neurons (Maekawa et al., 2013) and neuronal cell lines (Aung et al., 2013), and that inhibition of neuritogenesis by NaAsO_2_ is caused by alterations in the expression of cytoskeletal genes, tau, tubulin, and neurofilament (Aung et al., 2013), and suppression of glutamate AMPA receptor expression (Maekawa et al., 2013). Thus, inorganic arsenic adversely affects the fate and maturation processes of young neurons, which may lead to abnormal formation of neural circuits. In the present study, we found that the length of neurites in the PrL was significantly lower in the NaAsO_2_-exposed group, suggesting that prenatal exposure to NaAsO_2_ has an adverse effect on neuritogenesis. Elongation of the axon and dendrites is an essential event for the formation of basic neuronal circuitry. Impairments in the length and morphology of dendrites in the frontal cortex are involved in the pathogenesis of cognitive deficits and mental retardation (Armstrong et al., 1998). It indicates that the alteration in the morphology of neuron, particularly the PrL neuron, is strongly associated with the pathophysiological states of cognitive and learning dysfunction and that prenatal exposure to NaAsO_2_ may contribute to the pathogenesis. Additionally, it has been reported that the degree of learning disability is positively correlated with the severity and extent of dendritic abnormalities (Kaufmann and Moser, 2000). Therefore, we subsequently examined the density and morphology of pyramidal neuron dendritic spines in different layers of the PrL. In contrast to the impairment in neurite length, the morphology and the density of dendritic spines in PrL pyramidal neurons were not affected by NaAsO_2_ exposure. We recently demonstrated in cultured neurons that NaAsO_2_ specifically alters the gene expression of cytoskeletal proteins including tau, tubulin, and neurofilaments, but does not affect the expression of actin protein (Aung et al., 2013). Since dendritic spines are actin-rich protrusions from dendrites that form the post-synaptic component of a synapse (Hotulainen and Hoogenraad, 2010), our current finding that NaAsO_2_ exposure did not have an effect on synapse number agrees with our previous study regarding the expression of actin protein. NaAsO_2_ exposure has however been reported to impair the expression of AMPA and NMDA glutamate receptors (Maekawa et al., 2013; Ramos-Chavez et al., 2015), suggesting that NaAsO_2_ exposure can affect glutamate transmission. Because glutamate transmission is critically involved in the regulation of synapse formation (Rasse et al., 2005), we cannot exclude the possibility that the exposure to NaAsO_2_ alters synapse formation in areas other than the PrL. Taken together, the present study highlights the possible association between behavioral impairment in mice caused by prenatal NaAsO_2_ exposure and morphological alteration of brain, particularly cortical disarrangement in the prelimbic cortex.

On the other hand, the suggested association between the behavioral inflexibility and morphological alteration of the PrL was come from the morphometrical analysis, which was however carried out following the behavioral flexibility test. It has been demonstrated in human subjects that goal-directed learning is strongly associated with increase neural activity in prefrontal cortex (Valentin et al., 2007) and higher neurite density in medial orbitofrontal cortex (Morris et al., 2016). Since the control mice performed better than NaAsO_2_-exposed mice in this study of behavioral flexibility tasks, we could not deny the possibility of increase neurite length in the PrL of the control mice, which might be outcome of better goal-directed learning in behavioral flexibility tasks. In addition, it is important to note that the reduced maternal water consumption was observed in the group of dams provided with water containing NaAsO_2_, and the difference between the two groups was about 2 ml per day (Figure S5). It might be due to unpalatability of dams to water containing NaAsO_2_. Although we did not observe the obvious signs of maternal or embryonic toxicity such as maternal weight (Figure S1) and the number of pups (Figure S2) between the two groups of this study, several studies reported the possibility that maternal dehydration due to reduced water intake during pregnancy was associated with long-term physiologic effects on offspring such as development of brain function and plasma composition (Desai et al., 2005; Ross et al., 2005; Zhang et al., 2011). Therefore, we had to assume that behavioral inflexibility observed in mice prenatally exposed NaAsO_2_ could be induced by the combinatorial effect of the toxicity of prenatal NaAsO_2_ exposure and maternal dehydration.

In this study, we used male and female mice at 67 week (15.5 month) of their age in this study of behavioral flexibility tests, which additionally lasted for 10–12 weeks (about 2–3 month). Therefore, the age of mice in the last day of behavioral tests was being 17.5–18.5 month, which could be generally considered as old aged mice. It has been demonstrated that arsenic-induced increase in oxidative stress (such as glutathione level in the blood) was more prominent in young and old rats compared to adults (Jain et al., 2011, 2012). Motor impairments caused by prenatal arsenic exposure were observed in young juvenile mice, but such effects observed in young mice were subsided with advancing age (Markowski et al., 2012). These studies indicate age-dependent effects of arsenic-induced toxicity. Therefore, although we observed NaAsO_2_-induced behavioral inflexibility in old aged mice in the present study, we need further studies to test the age-dependent effects of prenatal NaAsO_2_ exposure on behavioral flexibility.

In conclusion, we demonstrate the possibility that *in utero* NaAsO_2_ exposure leads to behavioral inflexibility to changing tasks in adulthood, and cortical disarrangement in the PrL might contribute to this behavioral impairment. Further studies are required to elucidate how NaAsO_2_ disrupts neuronal development including axonal and dendritic elongation particularly in prefrontal cortex. Since behavioral inflexibility is observed in children with neurodevelopmental disorders such as autism spectrum disorders, our findings put forth a new perspective on how environmental exposures affect the pathogenesis of neurodevelopmental disorders.

## Author contributions

KA designed and performed experiments, analyzed data and wrote the paper; CT, KS performed experiments and analyzed data; KaN, AT, KeN, MK, and CT edited the paper; ST designed experiments and edited the paper; and FM designed experiments, analyzed data and wrote the paper.

### Conflict of interest statement

The authors declare that the research was conducted in the absence of any commercial or financial relationships that could be construed as a potential conflict of interest.
