# Supplementary figures and images for: Prenatal Exposure to Arsenic Impairs Behavioral Flexibility and Cortical Structure in Mice
Source: Front Neurosci. 2016 Mar 31;10:137. doi: 10.3389/fnins.2016.00137 (PMC4814721; doi:10.3389/fnins.2016.00137)

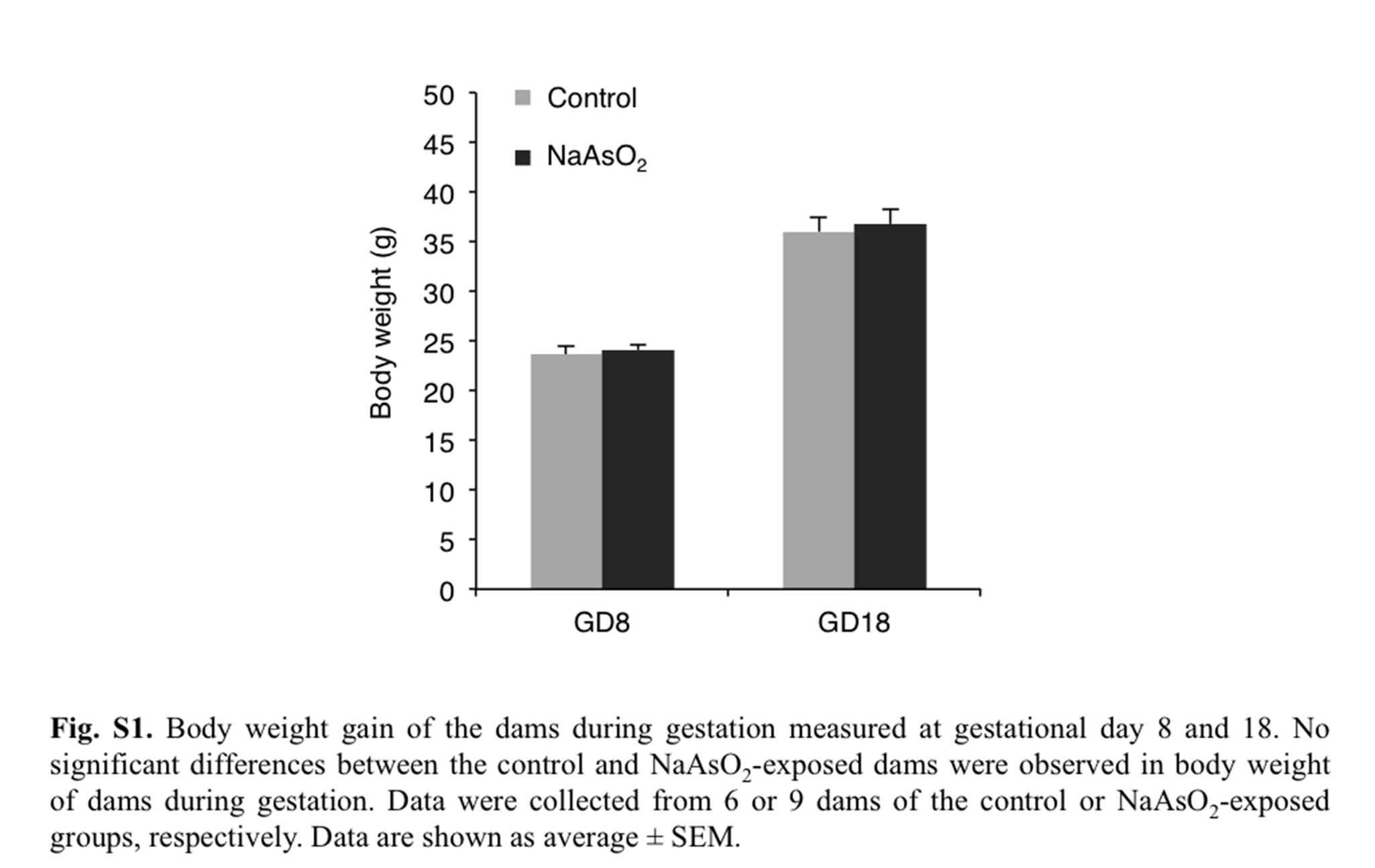

Supplement: Supplementary file 1 [file Image1.TIFF]

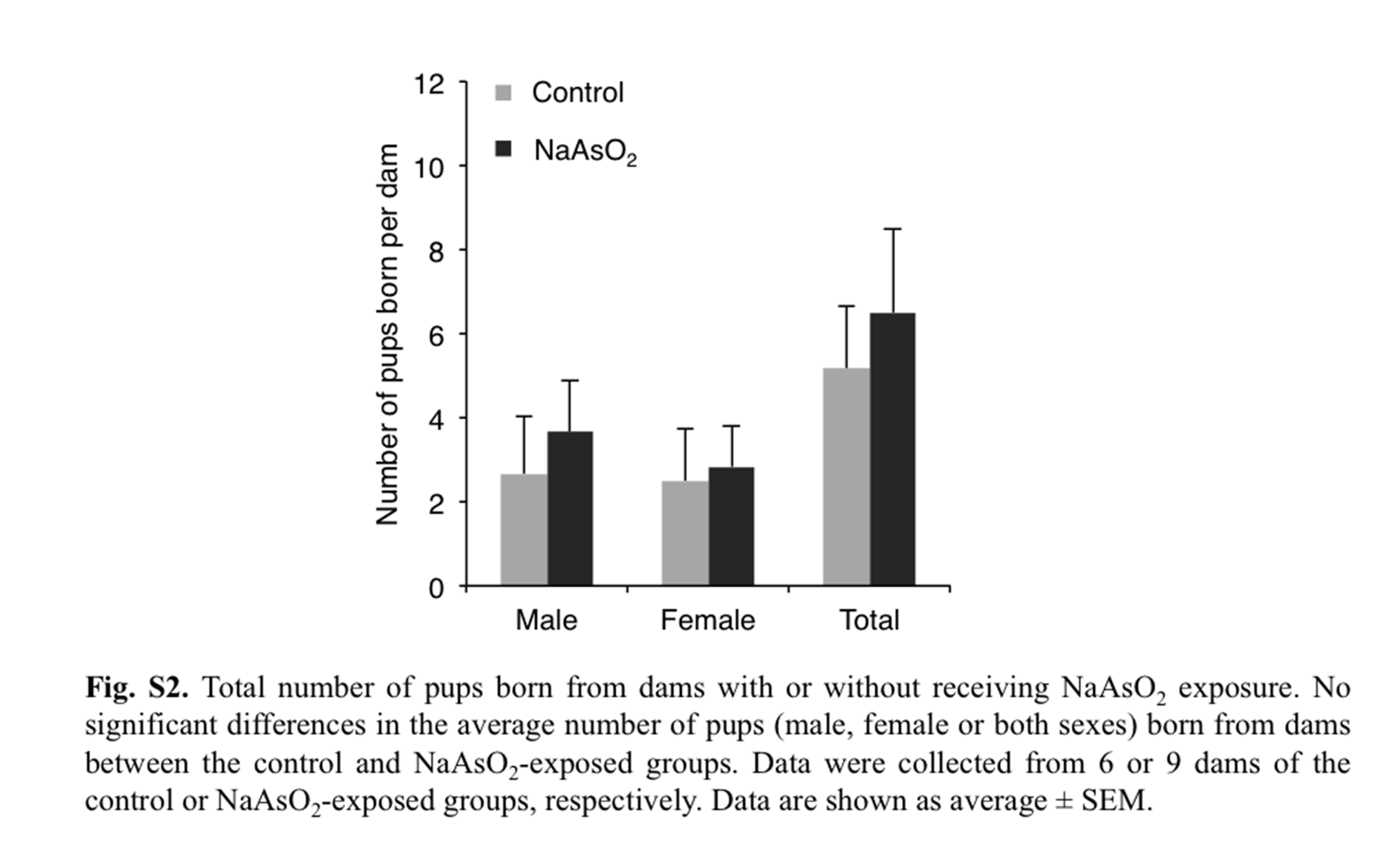

Supplement: Supplementary file 2 [file Image2.TIFF]

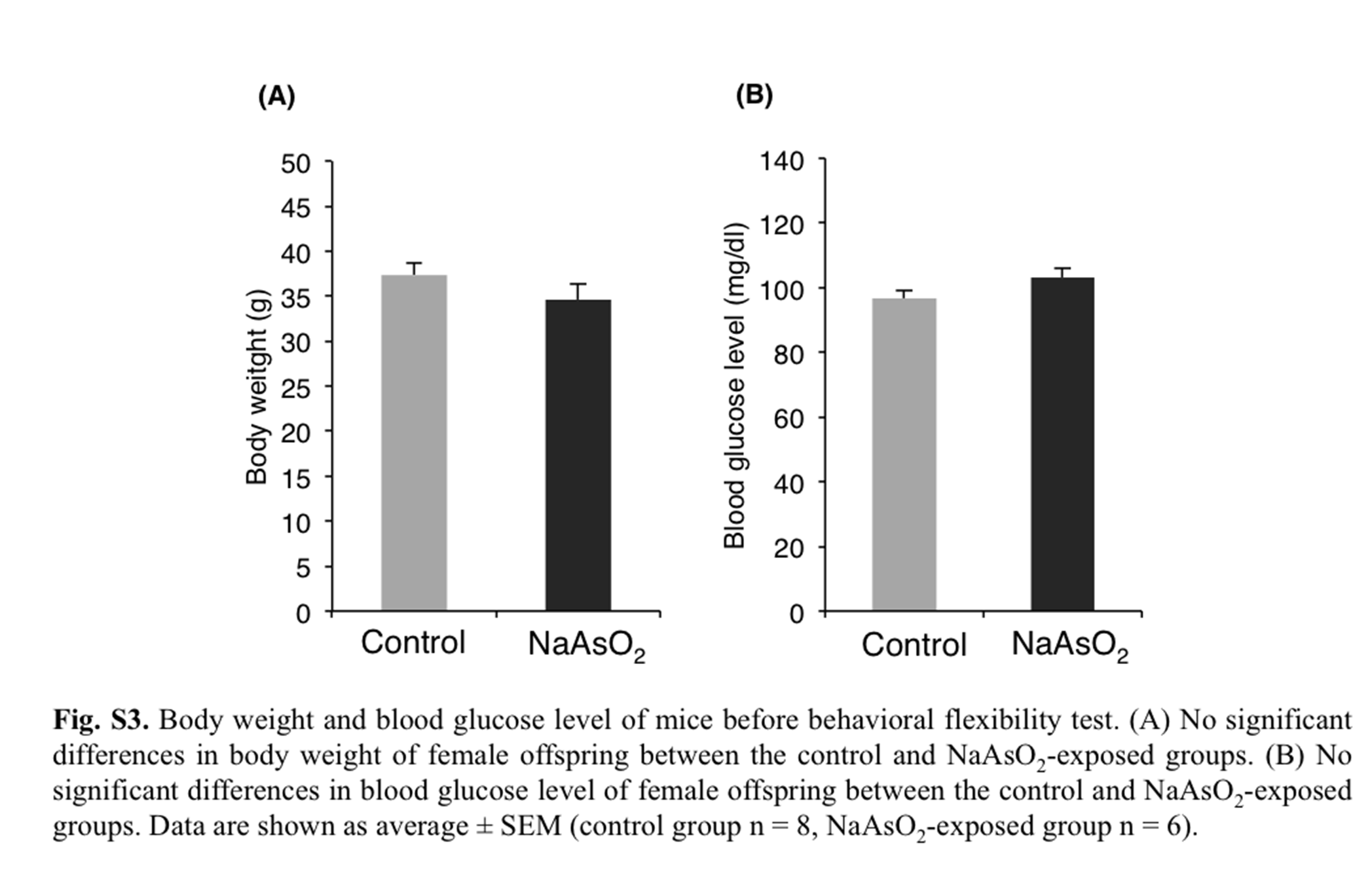

Supplement: Supplementary file 3 [file Image3.TIFF]

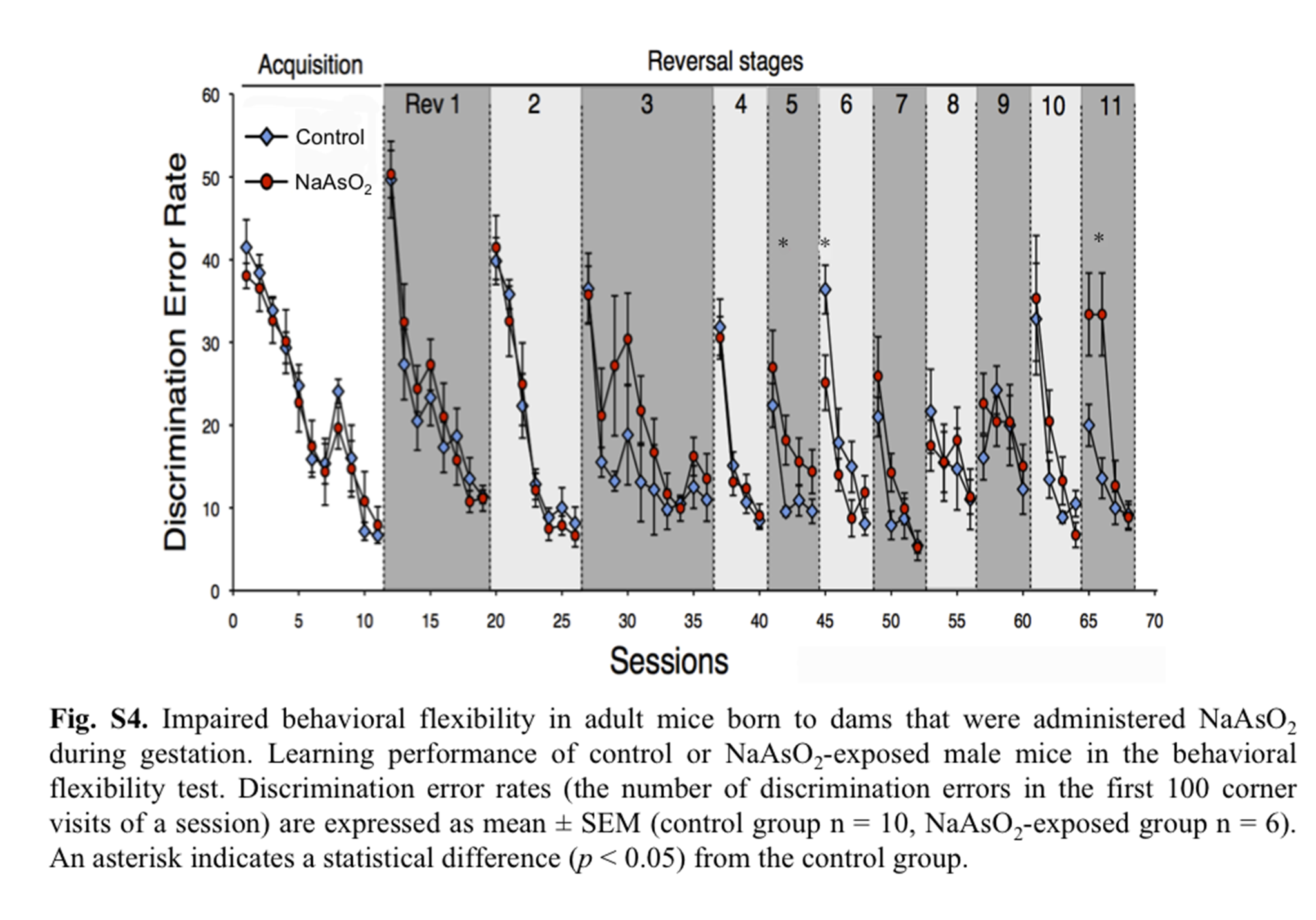

Supplement: Supplementary file 4 [file Image4.TIFF]

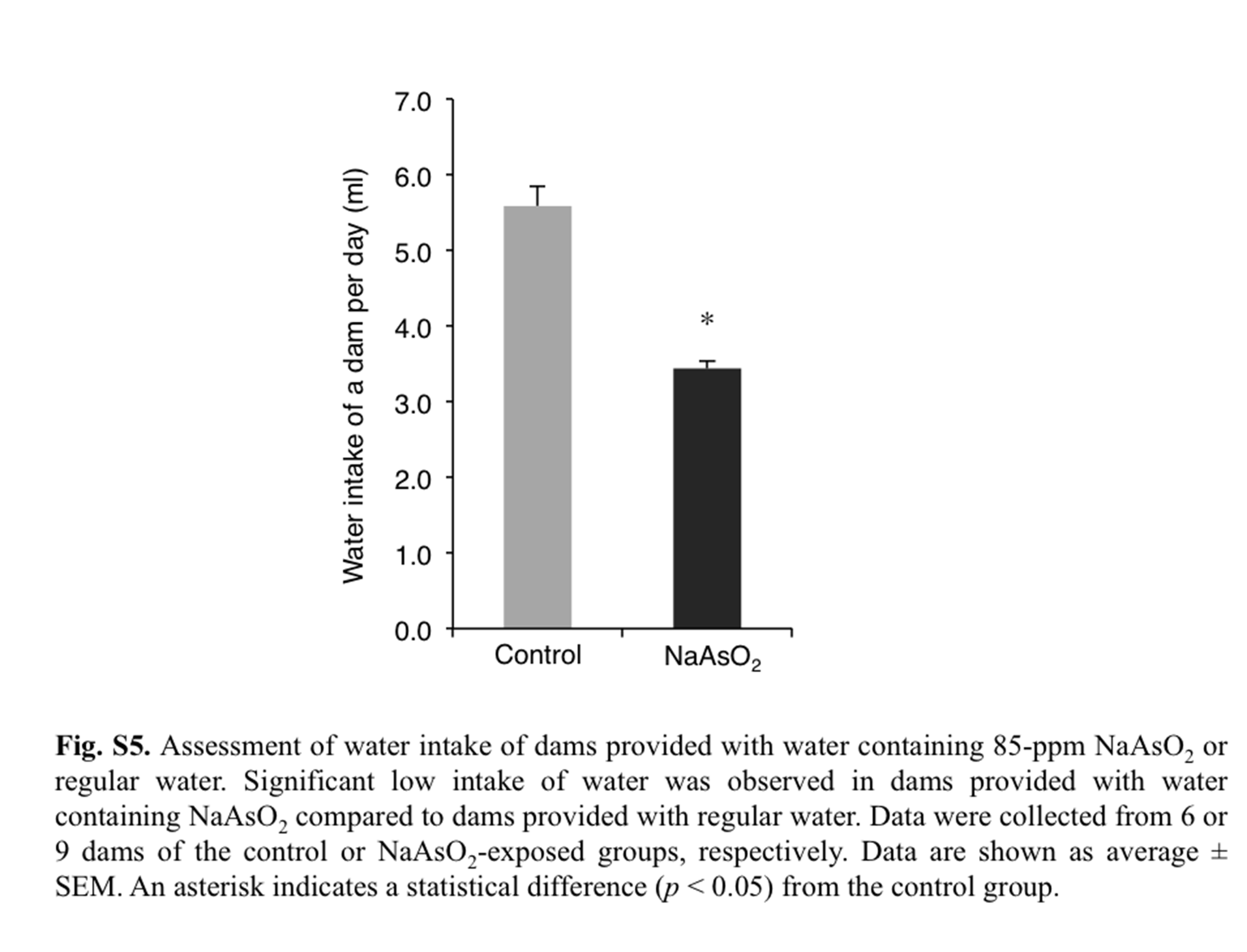

Supplement: Supplementary file 5 [file Image5.TIFF]
