# Supplementary material for: Prenatal Exposure to Arsenic Impairs Behavioral Flexibility and Cortical Structure in Mice
Source: Front Neurosci. 2016 Mar 31;10:137. doi: 10.3389/fnins.2016.00137 (PMC4814721; doi:10.3389/fnins.2016.00137)
Supplement: Supplementary file 6 [file Table1.DOCX]

Table S1. The exploratory and spontaneous activity indices of female mice extracted from the first 7 days of the acclimation phase of IntelliCage test (data are shown as average ± S.E.M.)

| Index | Control (n = 8) | NaAsO_2_ (n = 6) |
| --- | --- | --- |
| Total number of visits | 456 ± 43.0 | 636 ± 130.8 |
| Duration of visits (sec) | 9670 ± 594.6 | 8815 ± 351.7 |
| Total number of nose poke | 1253 ± 142.8 | 1204 ± 82.3 |
| Duration of nose poke (sec) | 3360 ± 163.4 | 4117 ± 257.6* |
| Total number of licking | 7734 ± 1044.9 | 9245 ± 1116.2 |
| Duration of licking (sec) | 1005 ± 121.3 | 1220 ± 108.7 |

*Significantly different from control group, *p* < 0.05. n = number of offspring randomly selected from dams (dam number: control = 6, NaAsO_2_ = 9)
