# Supplementary material for: Prenatal Exposure to Arsenic Impairs Behavioral Flexibility and Cortical Structure in Mice
Source: Front Neurosci. 2016 Mar 31;10:137. doi: 10.3389/fnins.2016.00137 (PMC4814721; doi:10.3389/fnins.2016.00137)
Supplement: Supplementary file 7 [file Table2.DOCX]

Table S2. The exploratory and spontaneous activity indices of male mice extracted from the first 7 days of the acclimation phase of IntelliCage test (data are shown as average ± S.E.M.)

| Index | Control (n = 6) | NaAsO_2_ (n = 10) |
| --- | --- | --- |
| Total number of visits | 382 ± 38.2 | 275 ± 37.6 |
| Duration of visits (sec) | 10065 ± 1148.1 | 9583 ± 977.9 |
| Total number of nose poke | 824 ± 162.4 | 821 ± 157.8 |
| Duration of nose poke (sec) | 7825 ± 909.1 | 5870 ± 363.8 |
| Total number of licking | 16299 ± 2620.8 | 16559 ± 1258.9 |
| Duration of licking (sec) | 2340 ± 347.3 | 2262 ± 137.6 |

n = number of offspring randomly selected from dams (dam number: control = 6, NaAsO_2_ = 9)
